# An Alkylphenol Mix Promotes Seminoma Derived Cell Proliferation through an ERalpha36-Mediated Mechanism

**DOI:** 10.1371/journal.pone.0061758

**Published:** 2013-04-23

**Authors:** Hussein Ajj, Amand Chesnel, Sophie Pinel, François Plenat, Stephane Flament, Helene Dumond

**Affiliations:** CNRS-Université de Lorraine, UMR 7039, Centre de Recherches en Automatique de Nancy, Vandoeuvre lès Nancy, France; University of Illinois at Chicago, United States of America

## Abstract

Long chain alkylphenols are man-made compounds still present in industrial and agricultural processes. Their main use is domestic and they are widespread in household products, cleansers and cosmetics, leading to a global environmental and human contamination. These molecules are known to exert estrogen-like activities through binding to classical estrogen receptors. *In vitro*, they can also interact with the G-protein coupled estrogen receptor. Testicular germ cell tumor etiology and progression are proposed to be stimulated by lifelong estrogeno-mimetic exposure. We studied the transduction signaling pathways through which an alkyphenol mixture triggers testicular cancer cell proliferation *in vitro* and *in vivo*. Proliferation assays were monitored after exposure to a realistic mixture of 4-tert-octylphenol and 4-nonylphenol of either TCam-2 seminoma derived cells, NT2/D1 embryonal carcinoma cells or testis tumor in xenografted nude mice. Specific pharmacological inhibitors and gene-silencing strategies were used in TCam-2 cells in order to demonstrate that the alkylphenol mix triggers CREB-phosphorylation through a rapid, ERα36-PI3kinase non genomic pathway. Microarray analysis of the mixture target genes revealed that this pathway can modulate the expression of the DNA-methyltransferase-3 (Dnmt3) gene family which is involved in DNA methylation control. Our results highlight a key role for ERα36 in alkylphenol non genomic signaling in testicular germ cell tumors. Hence, ERα36-dependent control of the epigenetic status opens the way for the understanding of the link between endocrine disruptor exposure and the burden of hormone sensitive cancers.

## Introduction

Over the last 50 years, the incidence of male reproductive disorders such as hypospadias, cryptorchidism, hypofertility and testis cancer has dramatically risen. For instance, testicular germ cell tumor (TGCT) has become the leading cause of cancer in men aged 15 to 45 years from industrialized countries. Among malignant tumors of the testis, 95% are testicular germ cell tumors, which are classified into two main categories based upon histologic, molecular and epigenetic traits: seminoma and nonseminoma [1]. Both derive from a common precursor cell type called carcinoma in situ (CIS) [2] which is believed to originate from misdifferentiated primordial germ cells or gonocytes in response to altered hormone signaling [3]. CIS cells appear during fetal life and then enter a period of dormancy in infancy until after puberty when TGCT emerge [4]. This prepubertal dormancy suggests a hormone sensitive mechanism for TGCT development and tumor progression at puberty. A wide range of published data dealing with TGCT geographic incidence variation, epidemiological studies performed on migrant men, and exposure model analyses *in vivo* and *in vitro* strongly suggest the participation of endocrine disrupting compounds (EDCs) in both initiation and progression of testis cancer. In occidentalized countries, this hypothesis also emerge to explain the burden of testis cancer associated pathologies such as cryptorchidism, hypospadias, hypofertility, as well as increased incidence of other hormone sensitive cancer (breast, prostate, ovary, endometrium) [5]–[8].

Among the great diversity of compounds potentially able to alter hormone signaling, plasticizers are of great concern because of (i) the ubiquitous and persistent environmental and human contamination, (ii) their presence in everyday life used cosmetics, food, drinking water, home cleansers and (iii) their ability to trigger estrogenic signaling [9].

These molecules such as bisphenol A (BPA), 4-nonylphenol (NP) or 4-tert-octylphenol (OP) belong to the alkylphenol family and are still used in various industrialized processes. Once released in the environment, they become persistent pollutants that are poorly eliminated by liver detoxification enzymes in mammals and can enter cells, especially in body fat due to their lipophilic properties [10], [11]. BPA and NP are also able to cross the seminiferous tubules basal lamina, alter the testis-blood barrier and trigger differentiating germ cell sloughing and apoptosis by disrupting Sertoli/germ cell attachment and communication [12]. Moreover, this class of EDCs appears to promote the development and progression of estrogen-dependent cancers [13]. BPA was also reported to promote mitogenic effect in JKT-1 seminoma derived cells [14], [15].

Binding experiments indicate that alkyphenols could mimic estrogen mitogenic signaling since BPA, NP and OP display a relative binding affinity to17β-estradiol (E_2_) in the range of 0.1% for the nuclear receptor ERα66 and 50% for the transmembrane g-protein coupled estrogen receptor (GPER) [9], [16]. Besides, nothing is known about alkylphenol binding to ERα36, a novel 36 kDa NH2-term truncated form of the canonical human estrogen receptor ERα66, retaining the DNA-binding, partial dimerisation and an altered ligand-binding domains [17]. ERα36 was previously shown by us and others to mediate estrogen non conventional mitogenic signaling in TCam-2 seminoma cells, triple-negative breast cancers cells and endometrial cancer cells [18]–[20]. Depending on the cell lines tested, ERα36 acts as a membrane located ER, sometimes collaborates with either GPER or EGFR, and triggers several kinase-dependent pathways, such as MAPK, STAT5 or PKA [20]–[23].

In an attempt to understand the mechanisms underlying the deleterious effects of EDCs on neoplastic germ cells, we aimed to decipher the alkylphenol-dependent transduction pathways in TCam-2 cells, a unique seminoma cell line [24]. The test compounds, 4-tert-octylphenol (4-t-OP) and 4-nonylphenol (4-NP) were mixed based on their realistic concentration ratio (1∶30) in food from Raecker and colleagues [25]. The resulting mix was called M4 and used at concentrations that mimic human environmental exposure. First, we show that M4 increases the TCam-2 seminoma cell proliferation rate *in vitro* by triggering the stimulation of ERα36 dependent mitogenic pathways. Second, we confirm the M4 stimulates NT2/D1 embryonal carcinoma cell proliferation in vitro as well as tumor growth in NT2/D1 xenografted nude mice. Finally, we demonstrate that alkylphenol signaling pathway ends on target genes involved in epigenetic modifications.

## Materials and Methods

### Reagents

The test compounds, 4-nonylphenol (CAS number: 84852-15-3), 4-tert-octylphenol (CAS no: 140-66-9), and 17β-estradiol (E_2_, CAS no: 50-28-2) were purchased from Sigma Aldrich (France). 4-tert-octylphenol (4-t-OP) and 4-nonylphenol (4-NP) were mixed based on their realistic concentration ratio (1∶30) in infant food [25], thus forming the working mix called M4. Stock solutions of 10 mM for M4 (the concentration refers to 4-NP therein) or E_2_ were prepared in dimethylsulfoxide (DMSO) and further diluted in RPMI medium without phenol red. All working solutions were freshly made just before the cell treatment assays.

Wortmanin (PI3K inhibitor), G1 (GPER agonist) and G15 (GPER antagonist) were purchased from Sigma-Aldrich (France). Control cells were treated with DMSO, diluted with the same factor as M4 and indicated as “vehicle” in the figures and ranging from 0.01% to 10^−12^%.

### Ethic Statement

Animals used in the present research have been treated humanely according to institutional guidelines (Directive EU/63/2010), with due consideration to the alleviation of distress and discomfort. Protocol for animal handling and experiments was approved by the “Lorraine Regional Committee for Animal Experiments” and carried out by competent and authorized persons (personal authorization number 54–89 issued by the Department of Vetenary Services) in a registered establishment (establishment number C-54-547-03 issued by the Department of Veterinary Services).

Animal experiment was planned in respect to the 3R rule: minimal number of mice necessary and sufficient to reach statistical significance was calculated *a priori*. For each animal, several organs (testis, liver, tumors, blood.) were harvested at the end of treatment for further analysis. Animals were housed in cages sized and filled with appropriate litter respectfully to European ethic guidelines, with free access to tap water and food *ad libidum*, in filtered atmosphere to avoid pathogen contamination.

Males were reared in individual cages to avoid fight stress and aggressiveness. Animals were housed for 3 weeks before the beginning of any experiment. Tumor grafts were performed rapidly under sodium pentobarbital anesthesia in a warm separate room. Tumor grafts, radiotherapy and resection were performed under general anesthesia All s.c. injections and animal handling were performed by the same technician in a separate room and all efforts were made to minimize suffering. At the end of treatment, mice were anesthetized with 8 mg/kg xylazine and 90 mg/kg ketamine injection, blood was collected by cardiac puncture and animals sacrificed by cervical dislocation.

### Nude Mouse Xenograft Model

Pathogen-free, 5–7 week-old male athymic NMRI-nu *(nu/nu)* mice were purchased from Janvier Laboratories (Le-Genest-St-Isle, France). Animals were housed in solid-bottomed plastic individual cages with free access to tap water and standard food *ad libidum*.

Primary tumors were obtained after intra-testicular injection of 1×10^7^ NT2/D1 cells. Six weeks later, tumors reached the ethic volume (0.5 cm^3^). Primary tumors were harvested and cut into 1 mm^3^ pieces in order to be grafted sub-cutaneously in Nude male mice.

Six males bearing bilateral NT2/D1 grafts were daily inoculated s.c. with either vehicle, or M4 (1.0 µg/kg bw or 10 µg/kg bw), 5 days per week. Nude mice were pre-treated for 2 weeks before graft, tumor pieces were grafted and treatment was applied for 4 additional weeks (Figure S1). The low dose was relevant to daily children intake (0.8 µg/kg bw/day) [25]. The tumor-take rate ranged from 95–100%. Mice weight (Figure S1) and tumor volume were monitored twice a week by caliper measurement of the length, width, and height and were calculated using the formula V = D*d^2^/2. At the end of treatment, tumors were removed, weighed, and fixed in 4% formalin for histological and immunohistochemical characterization (Figure S1).

### Cell Culture

TCam-2 and NT2/D1 cells were respectively maintained in RPMI1640 (GIBCO) and DMEM/F12 (1∶1, GIBCO) supplemented with 10% fetal calf serum (FCS, Invitrogen) and 2 mM L-glutamine and cultured in a 5% CO_2_ containing atmosphere at 37°C. Briefly, cells were plated in 10% FCS containing medium for 24 h and then starved for 24 h in 0.5% charcoal-stripped FCS-containing medium without phenol red. Treatments were performed on 0.5% charcoal-stripped FCS cultured cells, plated at a density of 8×10^4^ cells per well in 6-well plates. In case of inhibitor use, the corresponding compound was added to the medium 30 minutes before M4 or E_2_ treatment.

### Cell Proliferation Assay

Cells were seeded in 96-well plates at a density of 1×10^3^ cells/well, in 0.2 ml medium supplemented with 10% FCS and 2 mM L-glutamine. They were washed with PBS (GIBCO) once they had attached and then incubated in phenol red-free medium containing 0.5% charcoal-stripped FCS for 24 h. Cells were then submitted to the indicated treatments for 48 h. At the end of the treatment, cells were counted by using an inverted microscope. Each treatment was replicated six times. For proliferation assays, the DMSO dilution retained was similar to the one of M4 having the most important effect (1 nM M4 corresponding to 10^−5^% DMSO). However, none of the DMSO doses tested displayed proliferation stimulation effect compared to non treated cells.

### Real-time PCR Analysis

Reverse transcription and real-time PCR analyses were performed as previously described [19]. Large ribosomal protein (RPLPO) encoding gene was used as a control to obtain normalized values. Primers are listed in Table S1. Assays were performed at least in triplicate, and the mean values were used to calculate expression levels, using the ΔΔC(t) method referring to RPLPO housekeeping gene expression. When treatments were performed, the variation of expression was measured as treated/DMSO treated cells (control).

### RNA Interference

The small-interfering RNA (siRNA) duplexes for targeting GPER (Table S1) and scrambled control (SR-CL000-005) were purchased from Eurogentec (Angers, France). TCam-2 cells (8×10^4^) were plated into 6-well plates, in 2 ml of medium supplemented with 10% FCS and 2 mM L-glutamine the day before transfection**.** Cells were transiently transfected with either GPER (200 nM of the duplex) or scrambled siRNAs by using the Oligofectamine™ Reagent (Invitrogen) according to the manufacturer’s instructions. After 24 h, cells were washed with PBS and the medium was replaced with phenol red-free RPMI supplemented with 0.5% charcoal-stripped FCS and 2 mM L-glutamine. 24 h later, cells were treated in phenol red-free and 0.5% stripped FCS RPMI and harvested for further analyses. Efficacy of RNA interference is presented in Figure S2A.

### Western Immunoblotting

Western blot were performed as described previously [19]. The following primary antibodies were used: anti- ERα36 (diluted 1∶5000, Cell Applications, San Diego, USA), anti-DNMT3A (diluted 1∶500, Active Motif n°39897, La Hulpe, Belgium), anti-DNMT3B (diluted 1∶1000, Active Motif n°39899), anti-DNMT3L (diluted 1∶5000, Active Motif n°39907) and anti-phospho CREB (1∶1000; Epitomics #1113-5, Montrouge, France). The anti-alpha tubulin antibody (1∶5000, sc2004, Santa Cruz Biotechnology Inc., Santa Cruz, USA) was used as a control. The membranes were developed with ECL detection reagent using chemiluminescence (Amersham, Orsay, France). Protein quantification was performed using the Quantity One Geldoc software (Biorad, Marnes-la-Coquette, France).

### Transient Transfection and Establishment of Stable Cell Line

TCam-2 cells were transfected with the empty expression vector or the ERα36-specific shRNA expression vector kindly provided by Dr Wang ZY (Creighton University medical school, Omaha, USA) using the ExGen500 *in vitro* transfection reagent (Euromedex, France) as described previously [19]. Efficacy of shRNA knock-down is shown in Figure S2B.

### Statistical Analysis

Data were summarized as the mean ± s.e.m. Proliferation analysis data from each dose group were compared by analysis of variance (one-way ANOVA) followed by the Bonferroni multiple procedure with SPSS software (SPSS Inc., Chicago, USA). Real-time PCR and western blot experiments results were analyzed as follows: variance analysis of treated *versus* control cells was performed using Dunnett’s test for multiple comparisons. Differences in which *P* was less than 0.05 were considered as statistically significant.

## Results

### The M4 Alkylphenol Mix Stimulates Testicular Germ Cell Tumor Growth *in vitro* and *in vivo*

**Figure 1:**
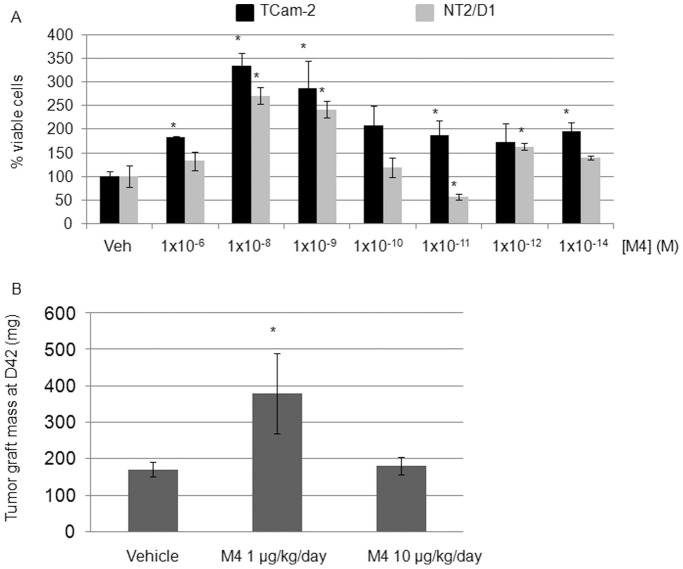
Alkylphenols stimulates TCam-2 and NT2/D1 cell proliferation *in vitro* and *in vivo*. (A) Cells were treated by M4 concentrations ranging from 1×10^−6^ to 1×10^−14^ M or vehicle for 48 h and counted under inverted microscope (see text for details). The DMSO dilution retained and indicated as “vehicle” was similar to the one of M4 having the most important effect: 1 nM M4 corresponding to 0.00001% DMSO. Notably, none of the DMSO doses tested (ranging from 0.01% to 10^−12^%) displayed proliferation modulating effect compared to non treated cells. Values indicated are the mean of 6 counts ±SEM. *P<0.05 *vs* vehicle treated. (B) Exposure to M4 alkylphenol mix stimulates tumor growth in nude male mice, injected 5 times a week with a M4 dose corresponding to human exposure or with a ten-fold higher dose. Tumors were weighed after a 6-week long treatment duration. Indicated values are the mean of at least 10 tumor weights ± SEM.

To test if an alkylphenol mix such as M4 could act as a proliferation inducer in seminoma-derived (TCam-2) and embryonal carcinoma (NT2/D1) cell lines, the growth rate of TCam-2 and NT2/D1 cells was determined by counting the number of cells exposed to different concentrations of M4. After a 24 h serum deprivation, cells were treated for 48 h with M4 decimal dilutions starting from 1.0 µM to 0.01 pM and counted by using an inverted microscope. M4 stimulated TCam-2 and NT2/D1 proliferation whatever the dose tested (Figure 1A). The dose-response curves of these cells to M4 exhibited non-monotonic or biphasic pattern. When compared to vehicle exposure, a maximum proliferation increase was observed for cells treated in the nanomolar range, which corresponds to environmental doses. Therefore, the dose of 1.0 nM was retained for further analyses.

To assess the effect of *in vivo* M4 exposure in male (androgenic; low estrogenic) hormonal context, a NT2/D1 derived germ cell tumor xenograft model was established (see material and methods section for details). Figure 1B shows that, at the end of treatment, tumor weight was significantly higher in M4 (1 µg/kg bw) *versus* vehicle injected mice. These data confirmed that an exposure to a low dose of M4, which corresponds to human daily intake [25] stimulates embryonal carcinoma growth *in vivo*. Notably, It is noteworthy that NT2/D1 cells knocked down for ERα36 are not viable. They can be selected and isolated after shRNA transfection but do not divide and therefore cannot be amplified for in vivo injection or used in vitro for proliferation assays. We also tried twice to obtain sh36-TCam-2 derived tumors after intra testicular injection of 1×10^6^, 5×10^6^, 1×10^7^ or 2×10^7^ cells but we never observed any tumor take, even 10 weeks after injection.

### M4 Triggers CREB Phosphorylation Through an ERα36 Dependent Pathway

**Figure 2:**
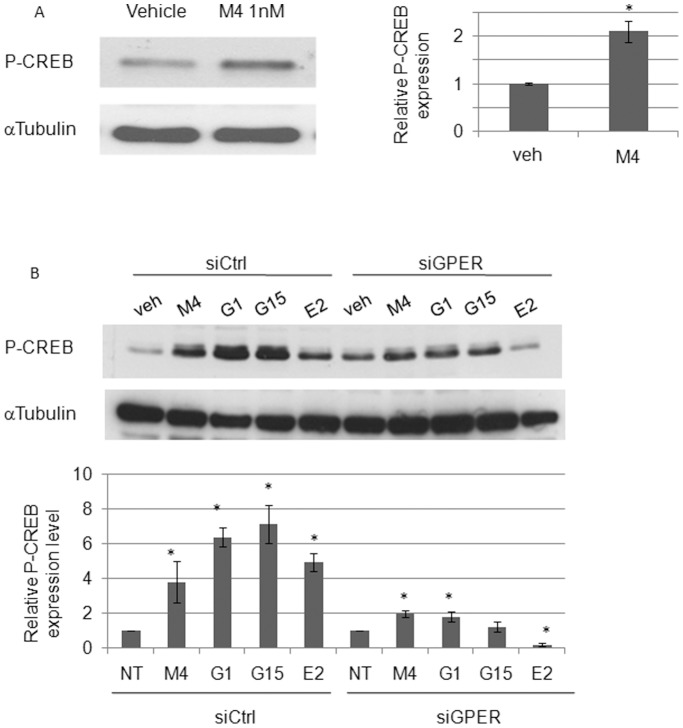
M4 induces CREB phosphorylation in TCam-2 cells through a GPER-independent pathway. (A) Cells were treated for 20 minutes with 1.0 nM of M4 and the level of phosphorylated CREB (P-CREB) was assessed by western-blot analyses compared to vehicle treated cells. (B) Western blot analyses of CREB phosphorylation upon exposure to 1.0 nM M4, 10 nM G1, 10 nM G15, or 1.0 nM E_2_ for 20 minutes in TCam-2 cells transfected by scrambled siRNA (siCtrl), or GPER-targeted siRNA (siGPER). Alpha tubulin stands for a loading control. Quantification means ± SEM and corresponding statistical analyses from at least three similar experiments are indicated. *P<0.05 *vs* vehicle treated.

Since we previously demonstrated that E_2_ and E_2_BSA both trigger CREB phosphorylation and in TCam-2 cells through GPER- ERα36 dependent mechanisms [19], we tested the potential estrogenicity of M4 by assessing phosphorylated CREB level. Western blot analysis clearly indicated an increase of CREB phosphorylation level (Figure 2A) after a 20 minute exposure to 1.0 nM M4. Several membrane receptors such as EGFR or GPER have been previously described to trigger estrogen-like signaling in various cancer cell lines [19], [26]. However, CREB phosphorylation induction was still observed in scrambled siRNA, EGFR-targeted transfected cells, suggesting that EGFR is not required for M4 signaling in TCam-2 seminoma-derived cells (data not shown). In order to check GPER involvement in M4 signaling, we used GPER agonists or antagonists in several contexts: scrambled siRNA transfected cells and their GPER-targeted counterparts were exposed to vehicle, 1.0 nM M4, 1.0 nM E_2_, 100 nM G1 (a GPER agonist) or 100 nM G15 (a GPER antagonist) for 20 minutes. M4, G1 and G15 appeared to be powerful inducers of CREB phosphorylation whereas E_2_ displayed lower efficiency in control cells (Figure 2B). In GPER-knocked down cells, M4, G1 and G15 could still stimulate CREB phosphorylation, even if this effect was milder, demonstrating that GPER activity was not fully required. As previously demonstrated, GPER knockdown prevented E_2_-dependent CREB phosphorylation, which suggests that the mechanisms involved in M4 signaling do not fully mimic those of estrogens [19]. Since G1 and G15 can act as ERα36 agonists and stimulate non genomic signaling pathways [21], [27] the results presented in Figure 2B also suggest that ERα36 in addition to GPER could trigger M4 dependent CREB phosphorylation.

### ERα36 Mediates M4 Induced Cell Proliferation and CREB Phosphorylation in TCam-2 Cells

**Figure 3:**
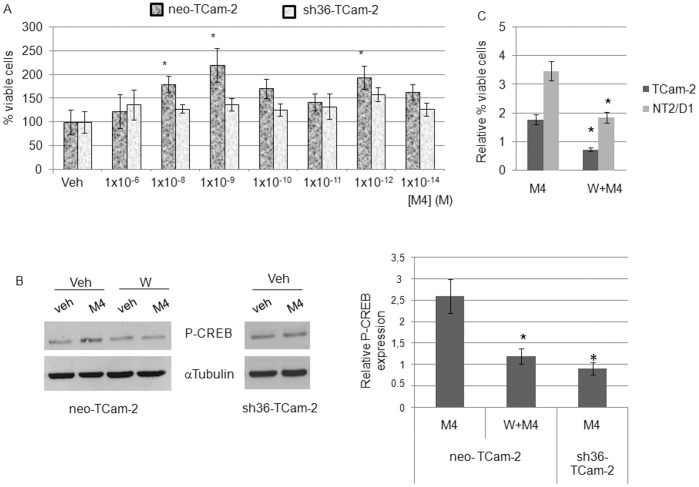
ERα36 mediates M4-induced PI3K-dependent proliferation and CREB-phosphorylation. (A) neo-TCam-2 or sh36-TCam-2 were previously described [-] : briefly, the neo-TCam-2 cell line was transfected with a control vector whereas sh36-TCam-2 stably expresses ERα36 targeted shRNA. Both cell lines were treated by M4 concentrations ranging from 1×10^−6^ to 1×10^−14^ M or vehicle for 48 h and cells were counted by using an inverted microscope. Values indicated are the mean of 6 counts ±SEM. *P<0.05 *vs* vehicle treated. (B) neo-TCam-2 or sh36-TCam-2 cell lines were treated for 20 minutes with 1.0 nM of M4 alone or after a 30 minute pre-treatment with 0.2 µM wortmanin. The level of phosphorylated CREB was then assessed by western-blot analysis and compared to vehicle treated cells. Quantification means ± SEM and corresponding statistical analyses from at least three similar experiments are indicated. *P<0.05 *vs* vehicle treated. (C) TCam-2 and NT2/D1 cells were pre-treated with 0.2 µM wortmanin for 1 hour before a 48 hour exposure to 1 nM M4. Cells were then counted by using an inverted microscope. Quantification means ± SEM. *P<0.05 *vs* M4 treated.

Normal human germ cells from the testis, malignant germ cells and their derived cell lines TCam-2 or NT2/D1 do not express the long form of ERα, ERα66. Nevertheless, they express the ERα36 isoform, which is necessary for mitogenic response to estrogens [19]. To address the hypothesis of ERα36 involvement in M4 proliferative effects, we performed dose-response experiments in the neo-TCam-2 and sh36-TCam-2 stable cell lines, which contain respectively, an empty plasmid or the corresponding vector expressing ERα36 targeted shRNA [19]. Figure 3A shows that a 48 h exposure to 1.0 nM M4 could stimulate neo-TCam-2 cell proliferation whereas this mitogenic effect was not observed in sh36-TCam-2 cells. Besides, CREB phosphorylation level did not increase in M4- treated ERα36-knocked down TCam-2 cells (Figure 3B). Both results strongly suggest that ERα36 is required for M4 signaling in seminoma-derived cells.

Neo-TCam-2 and sh36-TCam-2 cells were pre-treated with antagonists for several signaling pathways for 30 minutes before M4 exposure: a PKC inhibitor (BIM), the PKA inhibitor H89 and the PI3K inhibitor wortmanin. BIM did not prevent M4 induced CREB phosphorylation whereas H89 totally blocked it as well as its basal level, even in the non-M4 treated cells (data not shown). Wortmanin seemed to be the only antagonist able to prevent an M4 specific effect in neo-TCam-2, suggesting that M4 triggers ERα36-PI3kinase–dependent CREB phosphorylation in TCam-2 cells (Figure 3B).

The key role of PI3K-dependent signaling was further confirmed in both TCam-2 and NT2/D1 cells since wortmanin pre-treatment also impaired M4-enhanced cell proliferation (Figure 3C). Noteworthy, wortmanin, by itself, seemed to trigger a mild stimulation of NT2/D1 proliferation at high doses (1 µM to 10 µM; data not shown). Such an effect was not observed in TCam-2 cell line.

### M4 Represses DNA-methyltransferase Expression Through ERα36 Dependent Mechanisms in TCam-2 Cells

**Figure 4:**
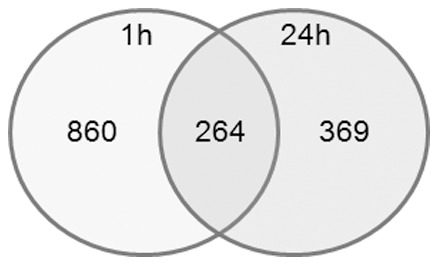
Venn diagram of M4 regulated genes as revealed by microarray analysis in TCam-2 cells. 860 genes and 369 genes were regulated by at least two-fold (p<0.05) after 1 hour or 24 hour 1 nM M4 treatment, respectively. 264 genes were commonly regulated at both exposure times.

In order to determine the transcriptional profile of TCam-2 cells exposed to M4, we performed a microarray analysis of gene expression after a 60 minutes or a 24 h treatment. TCam-2 cells were cultured for 24 h in 0.5% FCS containing medium in the presence of vehicle or 1.0 nM M4. Total RNA was extracted for global analysis of gene expression on Nimblegen microarray. Venn diagram presented in Figure 4 indicates that 1124 transcripts and 633 transcripts were up or down regulated (absolute variation factor ≥2 in duplicate RNA samples, *P*<0.05) after 1 hour or 24 hours M4 exposure, respectively. Among them, 264 genes were similarly regulated in both conditions and the corresponding list was analyzed for functional classes and networks with the Ingenuity software (Tables S2, S3, S4, [28]). As expected, main networks and biological functions associated to the list of M4 regulated genes referred to cancer, developmental disorder, cell growth and proliferation (Tables S2 and S3). Moreover, the predicted upstream regulators were all related to estrogens (Table S4).

**Figure 5:**
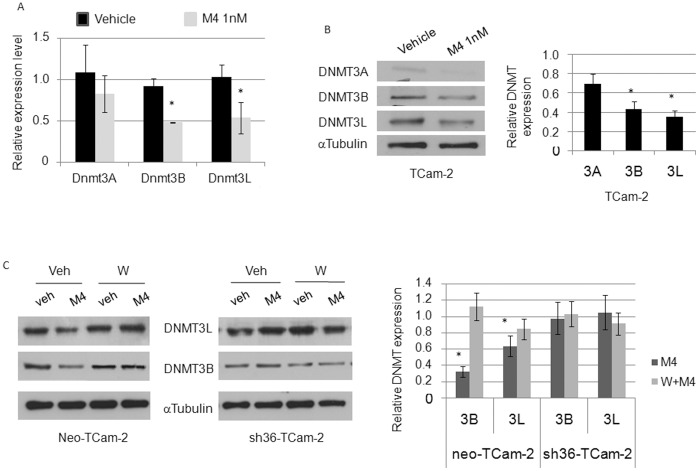
M4 represses the expression of Dnmt3A, Dnmt3B and Dnmt3L. (A–B). TCam-2 cells were treated for 24 hours with 1.0 nM of M4 and Dnmt3A, 3B or 3L expression was assessed by real-time PCR (A) or western-blot analysis (B). (C) M4-dependent Dnmt3B and Dnmt3L down-regulation was also observed in neo-TCam-2 cells but was impaired after a 30 minute pre-treatment with 0.2 µM wortmanin or in sh36-TCam-2 cells. Quantification means ± SEM and corresponding statistical analyses from at least three similar experiments are indicated. *P<0.05 *vs* vehicle treated.

**Figure 6:**
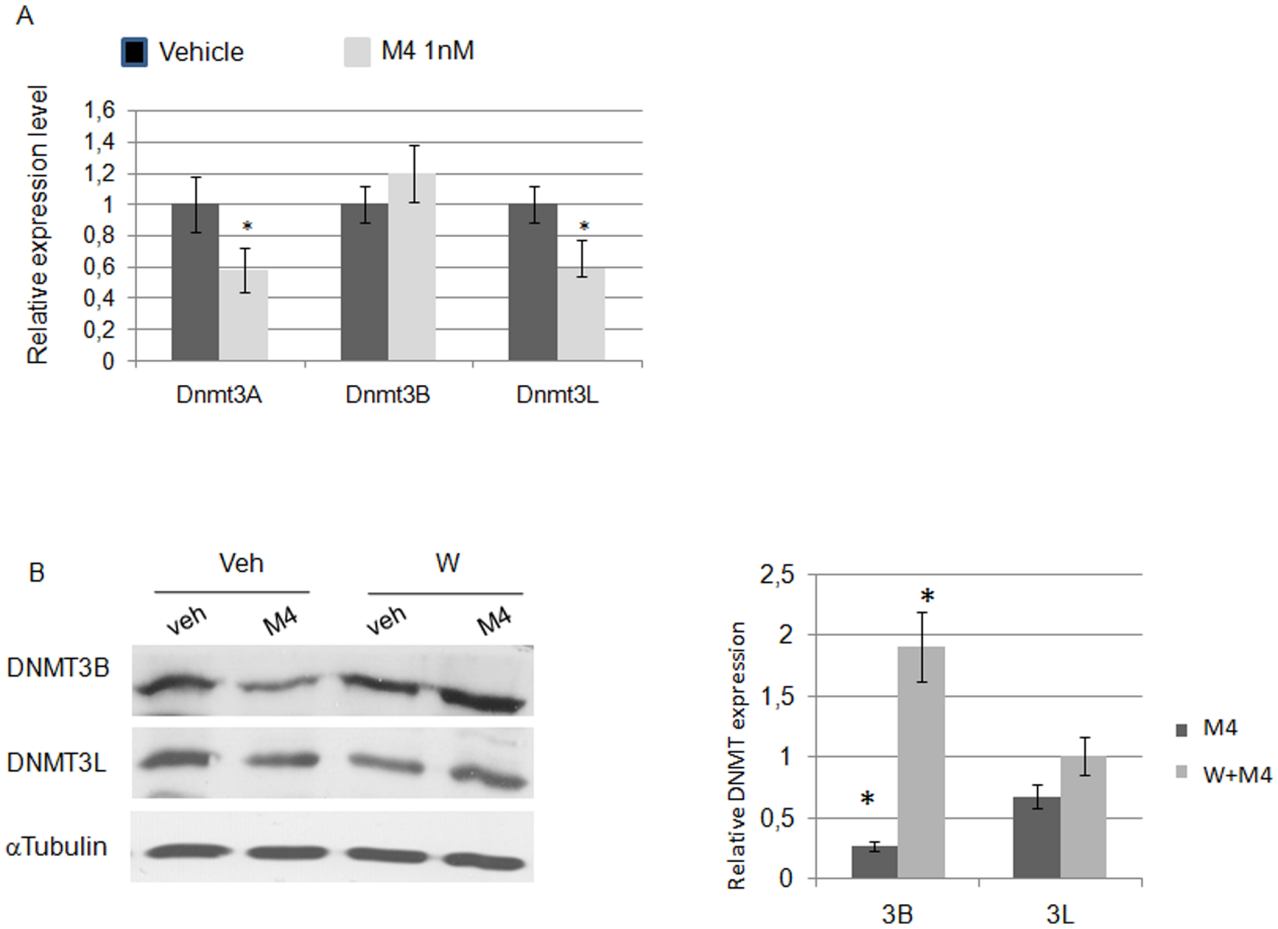
M4 modulates the expression of Dnmt3A, Dnmt3B and Dnmt3L through PI3K-dependent signaling. (A–B). NT2/D1 cells were treated with 1.0 nM of M4 and Dnmt3A, 3B or 3L expression was assessed by real-time PCR (A) or western-blot analysis without or after a 30 minute pre-treatment with 0.2 µM wortmanin (B). Quantification means ± SEM and corresponding statistical analyses from at least three similar experiments are indicated. *P<0.05 *vs* vehicle treated.

Among the functional classes of genes whose expression is significantly up- or down-regulated (top list provided in Table S5), we focused on those involved in epigenetic modifications which seemed related to PI3K/CREB and estrogen receptor signaling in Ingenuity sorting (Figure S3). Indeed, two of the three Dnmt3 genes display predicted CREB response elements half-sites (TGACG/CGTCA) in their promoter region (Dnmt3A: −684; −783; −1468; −2644; −3605; Dnmt3L:−2297; −2884 from the transcription start site) and therefore are good candidates for CREB-dependent expression control. Namely, the Dnmt3 gene family displayed a mild but reproducible down regulation after both 60 minutes and 24 h M4 exposures (Table 1). The results from microarray analysis were confirmed by quantitative RT-PCR (Figure 5A) and western-blot (Figure 5B) analyses. Indeed, M4 triggered a downregulation of DNMT3 expression in TCam-2 and neo-TCam-2 cells whereas such a repression was not observed in sh36-TCam-2 cells or after a 30 minute wortmanin pre-treatment (Figure 5C). Similar downregulation of Dnmt3A and Dnmt3L gene expression was observed in NT2/D1 cells (Figure 6A). Hence, M4- and PI3K-dependent repression of DNMT3B and DNMT3L was further observed at the protein level (Figure 6B). This suggests that the ERα36-dependent M4 signaling ending at target genes involved in DNA-methylation status could be a common feature of both seminoma and embryonal carcinoma cells.

**Table 1:** DNMT3 gene expression level variation after either 1 hour or 24 h M4 exposure as observed in microarray analysis in TCam-2 cells.

		1 h M4 exposure		24 h M4 exposure	
Sequence ID	Gene name	Fold	*p* value	Fold	*p* value
NM_175630	DNMT3A	−2.25	0.03	−2.16	0.01
NM_006892	DNMT3B	−2.65	0.01	−3.19	0.01
NM_013369	DNMT3L	−2.70	0.001	−2.96	0.001

## Discussion

Although numerous chemicals are now known or suspected to have endocrine disruption effects, a relevant classification based on comprehensive understanding of their mode of action and targets is still failing. More confusing is the wide variety of cocktails detected in the environment, when trying to decipher dose–response consequences for lifelong human exposure. In the present study, we chose to focus on a well defined mix of alkyphenols - M4 -composed of 4-tert-octylphenol and 4-nonylphenol (1∶30 ratio). Despite the burden of recent research on BPA which belongs to the same chemical family and exert various estrogenic effects, 4-tert-octylphenol and 4-nonylphenol are still neglected. High doses of tert-octylphenol or nonylphenol ranging from 25 to 200 mg/kg bw were previously shown to significantly decreased sperm count and quality in male mice, and affect uterine weight, vaginal opening and reproductive ability in female rats [29], [30]. However, both molecules have never been associated in a realistic mixture mimicking daily human contamination from household products, cosmetics and food. Here, estrogen-like mechanisms of action were addressed in a model of TGCT lacking the long form of ERα receptor (ERα66). As in the case of 17β-estradiol or its BSA-coupled counterpart, M4 doses ranging from 10 nM to 0.1 nM stimulated both seminoma and embryonal carcinoma cell proliferation in a non monotonic dose-response manner. Moreover, we observed a positive impact of M4 exposure on tumor growth in a TGCT xenograft nude mouse model after a treatment corresponding to human intake (1 µg/kg bw) [25]. No stimulating effect was detected after exposure to the higher dose (10 µg/kg bw), suggesting (i) that tumor growth in xenografted mice could respond to M4 in a non-monotonic way, as observed for in vitro cell proliferation or (ii) that a mild toxicity could appear after exposure to high doses of the mix. Taken together, these results suggest that alkylphenol exposure may on the one hand, alter normal germ cell multiplication and differentiation during development [12], [31] through mutagenic or clastogenic mechanisms at high doses [32], [33] as described by others and on the other hand, elicit neoplastic germ cell proliferation at low doses, as shown in this study.

Therefore we investigated the rapid non-genomic transduction pathways potentially involved. Whereas estradiol and BPA were previously shown to bind and exert such mitogenic effects through GPER in both SKBR3 breast cancer cells and JKT-1 seminoma derived cells [15], [34], we demonstrated that M4 acts mainly via an ERα36-dependent pathway. Indeed, we evidence here that M4 triggers PI3K activity and CREB phosphorylation. Nevertheless, preliminary data indicate that both GPER and ERα36 may activate downstream signaling such as src phosphorylation and thus modulate the expression of M4 target gene subclasses as well as cell proliferation (A. Chesnel, personal communication). Since GPER was shown to partially govern ERα36 expression in our TCam-2 model [19] and may collaborate with ERα36 for estrogenic activities in other cancer cell lines [26], [35], it would be relevant to test the participation of ERα36 in alkylphenol response in hormone-sensitive cancers such breast or prostate cancers.

The microarray analysis performed in order to describe the gene expression pattern of M4-treated TCam-2 cells, indicated that several epigenetic modification enzymes encoding genes are affected. We focused on M4-dependent down-regulation of DNMT3 expression because (i) other estrogeno-mimetic such as genistein and resveratrol or anti-androgenic compounds such as vinclozolin that are present in food have been previously demonstrated to modulate tumor suppressor gene expression through epigenetic mechanisms [36], [37], (ii) DNMT3 proteins have been shown to be involved in germ cell proliferation and differentiation control during a developmental window when neoplastic germ cells (CIS) are believed to emerge [38], (iii) polymorphism of these genes is clearly associated with gastric and breast cancer, as well as ovarian endometriosis susceptibility [39]–[41]. Indeed, “Ingenuity sorting” clearly classifies DNMT3 downregulation into functional networks involved in cancer progression and cell proliferation downstream estrogen receptors and estrogens (Tables S2, S3, S4). Moreover, several studies point out the key role of DNA methylation in testicular tumor initiation, progression and resistance to chemotherapy [42]–[46], highlighting the importance for examining carefully which upstream compounds or regulation factors are able to modulate DNMT expression and activity.

Hence, our results indicate that either wortmanin treatment or ERalpha36 knockdown can impair M4-dependent Dnmt3 repression while ERalpha36 expression appears to be necessary for M4-dependent enhanced proliferation.

CREB target gene database detects CREB response elements half-sites in Dnmt3A and Dnmt3L promoters and further suggest that both gene could be a target for the PI3K/CREB dependent pathway [47]. Moreover, Hervouet and coworkers [48] demonstrated that DNMT3B and 3A can physically interact with several transcription factors involved in proliferation control, such as CREB, FOSB, KLF12, EGR1 or JUN, which were proposed to direct methylation on specific gene promoter sequences. DNMT3 can also regulate each other expression through promoter methylation [38].

Finally, since ERalpha36 promoter is located into the first intron of ESR1 gene, balanced expression of either ERalpha66 or ERalpha36 could be regulated by differential methylation. This point was already addressed by others who demonstrated that downregulation of DNMT3A and DNMT3B led to regulation of ESR1 or ESR2 via promoter DNA aberrant methylation in acute myeloid leukemia, endometriosis, prostate and ovarian cancer [49]–[52].

Endocrine disruptors such as alkylphenols are also suspected to alter germ cell epigenetic reprogramming during fetal and perinatal development, thus triggering long-term disruption of gene expression which, in turn, could be a main risk factor for hormone-dependent cancers. Anway and colleagues [37] also found DNMT3A and DNMT3L isoforms to be repressed in the testis after embryonic exposure to the endocrine disruptor vinclozolin. This commonly used fungicide suspected to have antiandrogenic effects triggered transgenerational epigenetic reprogramming associated with increased adult onset diseases, namely prostate disease, testis abnormalities, and tumor development [53]. Therefore, it could be relevant to address the effects of delayed consequences of a M4 exposure.

Surprisingly, DNMT3L expression was clearly detected at both mRNA and protein level in TCam-2 seminoma-derived cells by using commercially available antibody contrary to previous work on human biopsies using homemade polyclonal antibody and indicating that DNMT3L expression was restricted to embryonal carcinoma [54]. In the germ cell lineage, DNMT3L is involved in *de novo* retrotransposon methylation and appears to be a signature of prospermatogonia stage [55]. Therefore, the expression of DNMT3L could be a hallmark of undifferenciated stage.

DNMT3A and DNMT3B are usually described as enzymes responsible for the establishment of specific CpG dinucleotides methylation essential for embryonic development and gene repression at the time of implantation [56]. However, a growing number of evidences support the hypothesis for their contribution in the maintenance of DNA methylation [57]. DNMT3L also participates in a complex coupling H3K4 methylation and DNMT3A-dependent DNA methylation, thus modifying chromatin accessibility [58]. Therefore, M4-dependent downregulation of these enzymes could trigger DNA hypomethylation and chromatin opening, thus leading to aberrant gene expression pattern, which can be maintained transgenerationally. These observations are of particular interest in the field of testicular germ cell carcinogenesis since CIS are proposed to originate in non-differentiated primordial germ cells displaying a gene expression pattern of pluripotency. This could be also relevant in the context of testis tumor growth since lifelong M4 exposure may maintain a population of non-differentiated/highly proliferative cancer cells in the tumor.

Taken together, these data suggest that M4 exposure may elicit a positive feedback loop beginning at ERα36 activation, triggering PI3K-dependent CREB phosphorylation, ending on Dnmt3 repression which, in turn, could stimulate and maintain a high level of ERα36 expression and rapid proliferation. Epigenetic regulation of ESR1 locus remains to be carefully examined in various cell contexts in order to address such a hypothesis.

## Supporting Information

Figure S1 Characterization of testis tumor xenograft model in nude mice. Germ cell tumor xenograft models were first established after intra testicular injection of 1×10^7^ TCam-2 or NT2/D1 cells in 0,9% NaCl in nude mice. Tumors developed to approximately 0.5 cm^3^ in 6 weeks. MRI imaging was used (Spectro-imageur Bruker Biospec Avance 24/40; 2.4 teslas magnetic field) to confirm the presence of tumors into the scrotum. Tumors were harvested and seminoma or embryonal carcinoma identity was attested by histological and immunohistochemical analyses. The slices presented are hematoxylin/eosin/safran colorations of TCam-2 derived or NT2/D1 derived tumors. Tumor tissue was harvested and subcutaneously (s.c.) grafted in the inguinal pit of male nude mice. Because NT2/D1, but not TCam-2 derived tumors developed, we focused on the NT2/D1 model to examine the effects of M4 on tumor growth. Nude mice were s.c. implanted with 1–2 mm^3^ tumor pieces harvested from previously grown (0.5 cm^3^) NT2/D1 tumor (third passage). For alkylphenol assay, M4 or vehicle treatment was injected five days per week subcutaneously in male nude mice 2 weeks before, and 4 weeks after tumor graft in order to mimic everyday life contamination (see text for details). Bars are 100 µm long.
(TIF)

Figure S2 Real time PCR analysis (A) and western blot (B) showing the efficacy of GPER- or ERα36 -silencing in TCam-2 cells, respectively.
(TIF)

Figure S3 General scheme of Ingenuity software analysis showing the regulation network which involves DNMT3 gene family.
(TIF)

Table S1 Primer and siRNA sequences.
(DOCX)

Table S2 Main results from Ingenuity analysis: top five networks in which M4 regulated genes are involved.
(DOCX)

Table S3 Main results from Ingenuity analysis: biological functions in which M4 regulated genes are involved.
(DOCX)

Table S4 Main results from Ingenuity analysis: Top five predicted upstream regulators of M4 regulated genes.
(DOCX)

Table S5 Top list of the 20 genes deregulated by M4 after 1 hour or 24 hour exposure.
(DOCX)

## References

[1] Vega A, Baptissart M, Caira F, Brugnon F, Lobaccaro JM, et al (2012) Epigenetic, a molecular link between testicular cancer and environmental exposures. Front Endocrinol (Lausanne) 3: 150 doi: 10.3389/fendo.2012.00150. 23230429 10.3389/fendo.2012.00150 PMC3515880

[2] Skakkebaek NE (1975) Atypical germ cells in the adjacent “normal” tissue of testicular tumours. Acta Pathol Microbiol Scand A 83: 127–130. 1124645 10.1111/j.1699-0463.1975.tb01364.x

[3] Sonne SB, Almstrup K, Dalgaard M, Juncker AS, Edsgard D, et al (2009) Analysis of gene expression profiles of microdissected cell populations indicates that testicular carcinoma in situ is an arrested gonocyte. Cancer Res 69: 5241–5250. 19491264 10.1158/0008-5472.CAN-08-4554 PMC2869030

[4] Rajpert-De Meyts E, Hoei-Hansen CE (2007) From gonocytes to testicular cancer: the role of impaired gonadal development. Ann NY Acad Sci 1120: 168–180. 18184914 10.1196/annals.1411.013

[5] Beiki O, Granath F, Allebeck P, Akre O, Moradi T (2010) Subtype-specific risk of testicular tumors among immigrants and their descendants in Sweden, 1960 to 2007. Cancer Epidemiol Biomarkers Prev 19: 1053–1065. 20354126 10.1158/1055-9965.EPI-09-1190

[6] Cyriac S, Rajendranath R, Louis AR, Sagar TG (2012) Familial germ cell tumor. Indian J Hum Genet 18: 119–121. 22754236 10.4103/0971-6866.96679 PMC3385167

[7] Main KM, Skakkebaek NE, Virtanen HE, Toppari J (2010) Genital anomalies in boys and the environment. Best Pract Res Clin Endocrinol Metab 24: 279–289. 20541152 10.1016/j.beem.2009.10.003

[8] Skakkebaek NE, Rajpert-De Meyts E, Main KM (2001) Testicular dysgenesis syndrome: an increasingly common developmental disorder with environmental aspects. Hum Reprod 16: 972–978. 11331648 10.1093/humrep/16.5.972

[9] Thomas P, Dong J (2006) Binding and activation of the seven-transmembrane estrogen receptor GPR30 by environmental estrogens: a potential novel mechanism of endocrine disruption. J Steroid Biochem Mol Biol 102: 175–179. 17088055 10.1016/j.jsbmb.2006.09.017

[10] Calafat AM, Kuklenyik Z, Reidy JA, Caudill SP, Ekong J, et al (2005) Urinary concentrations of bisphenol A and 4-nonylphenol in a human reference population. Environ Health Perspect 113: 391–395. 15811827 10.1289/ehp.7534 PMC1278476

[11] Müller S, Schmid P, Schlatter C (1998) Pharmacokinetic behavior of 4-nonylphenol in humans. Environ Toxicol Pharmacol 5: 257–265. 21781872 10.1016/s1382-6689(98)00009-x

[12] McClusky LM, de Jager C, Bornman MS (2007) Stage-related increase in the proportion of apoptotic germ cells and altered frequencies of stages in the spermatogenic cycle following gestational, lactational, and direct exposure of male rats to p-nonylphenol. Toxicol Sci 95: 249–256. 17065434 10.1093/toxsci/kfl141

[13] Fucic A, Gamulin M, Ferencic Z, Katic J, Krayer von Krauss M, et al (2012) Environmental exposure to xenoestrogens and oestrogen related cancers: reproductive system, breast, lung, kidney, pancreas, and brain. Environ Health 11 Suppl 1S8. 22759508 10.1186/1476-069X-11-S1-S8 PMC3388472

[14] Bouskine A, Nebout M, Brücker-Davis F, Benahmed M, Fenichel P (2009) Low doses of bisphenol A promote human seminoma cell proliferation by activating PKA and PKG via a membrane G-protein-coupled estrogen receptor. Environ Health Perspect 117: 1053–1058. 19654912 10.1289/ehp.0800367 PMC2717129

[15] Chevalier N, Vega A, Bouskine A, Siddeek B, Michiels JF, et al (2012) GPR30, the non-classical membrane G protein related estrogen receptor, is overexpressed in human seminoma and promotes seminoma cell proliferation. PLoS One 7: e34672. 22496838 10.1371/journal.pone.0034672 PMC3319601

[16] Li Z, Zhang H, Gibson M, Li J (2012) An evaluation on combination effects of phenolic endocrine disruptors by estrogen receptor binding assay. Toxicol In Vitro 26: 769–774. 22692143 10.1016/j.tiv.2012.05.017

[17] Wang Z, Zhang X, Shen P, Loggie BW, Chang Y, et al (2005) Identification, cloning, and expression of human estrogen receptor-alpha 36, a novel variant of human estrogen receptor-alpha 36. Biochem Biophys Res Commun 336: 1023–1027. 16165085 10.1016/j.bbrc.2005.08.226

[18] Tong JS, Zhang QH, Wang ZB, Li S, Yang CR, et al (2010) ER-α36, a novel variant of ER-α, mediates estrogen-stimulated proliferation of endometrial carcinoma cells via the PKCδ/ERK pathway. PLoS One 5: e15408. 21079811 10.1371/journal.pone.0015408 PMC2973969

[19] Wallacides A, Chesnel A, Ajj H, Chillet M, Flament S, et al (2012) Estrogens promote proliferation of the seminoma-like TCam-2 cell line through a GPER-dependent ERalpha induction. Mol Cell Endocrinol 350: 61–71. 22138413 10.1016/j.mce.2011.11.021

[20] Zhang XT, Ding L, Kang LG, Wang ZY (2012) Involvement of ER-α36, Src, EGFR and STAT5 in the biphasic estrogen signaling of ER-negative breast cancer cells. Oncol Rep 27: 2057–2065. 22426783 10.3892/or.2012.1722 PMC3380083

[21] Kang L, Zhang X, Xie Y, Tu Y, Wang D, et al (2010) Involvement of estrogen receptor variant ER-alpha36, not GPR30, in nongenomic estrogen signaling. Mol Endocrinol 24: 709–721. 20197310 10.1210/me.2009-0317 PMC2852353

[22] Wang L, Wang ZY (2010) The Wilms’ tumor suppressor WT1 induces estrogen-independent growth and anti-estrogen insensitivity in ER-positive breast cancer MCF7 cells. Oncol Rep 23: 1109–1117. 20204298 10.3892/or_00000739 PMC2837513

[23] Zhang XT, Kang LG, Ding L, Vranic S, Gatalica Z, et al (2011) A positive feedback loop of ER-α36/EGFR promotes malignant growth of ER-negative breast cancer cells. Oncogene 30: 770–780. 20935677 10.1038/onc.2010.458 PMC3020987

[24] Mizuno Y, Gotoh A, Kamidono S, Kitazawa S (1993) Establishment and characterization of a new human testicular germ cell tumor cell line (TCam-2). Nihon Hinyokika Gakkai Zasshi 84: 1211–1218. 8394948 10.5980/jpnjurol1989.84.1211

[25] Raecker T, Thiele B, Boehme RM, Guenther K (2011) Endocrine disrupting nonyl- and octylphenol in infant food in Germany: considerable daily intake of nonylphenol for babies. Chemosphere 82: 1533–1540. 21185059 10.1016/j.chemosphere.2010.11.065

[26] Prossnitz ER, Barton M (2011) The G-protein-coupled estrogen receptor GPER in health and disease. Nat Rev Endocrinol 7 715–726. 21844907 10.1038/nrendo.2011.122 PMC3474542

[27] Wang Z, Zhang X, Shen P, Loggie BW, Chang Y, et al (2006) A variant of estrogen receptor-{alpha}, hER-{alpha}36: transduction of estrogen- and antiestrogen-dependent membrane-initiated mitogenic signaling. Proc Natl Acad Sci USA 103: 9063–9068. 16754886 10.1073/pnas.0603339103 PMC1482566

[28] Long F, Liu H, Hahn C, Sumazin P, Zhang MQ, et al (2004) Genome-wide prediction and analysis of function-specific transcription factor binding sites. In Silico Biol 4: 395–410. 15506990

[29] Laws SC, Carey SA, Ferrell JM, Bodman GJ, Cooper RL (2000) Estrogenic activity of octylphenol, nonylphenol, bisphenol A and methoxychlor in rats. Toxicol Sci 54: 154–167. 10746942 10.1093/toxsci/54.1.154

[30] Willoughby KN, Sarkar AJ, Boyadjieva NI, Sarkar DK (2005) Neonatally administered tert-octylphenol affects onset of puberty and reproductive development in female rats. Endocrine 26: 161–168. 15888928 10.1385/ENDO:26:2:161

[31] Kilian E, Delport R, Bornman MS, de Jager C (2007) Simultaneous exposure to low concentrations of dichlorodiphenyltrichloroethane, deltamethrin, nonylphenol and phytoestrogens has negative effects on the reproductive parameters in male Spraque-Dawley rats. Andrologia 39: 128–135. 17683461 10.1111/j.1439-0272.2007.00777.x

[32] Dobrzyńska MM (2012) Male-mediated F1 effects in mice exposed to nonylphenol or to a combination of X-rays and nonylphenol. Drug Chem Toxicol 35: 36–42. 21848393 10.3109/01480545.2011.586036

[33] Frassinetti S, Barberio C, Caltavuturo L, Fava F, Di Gioia D (2011) Genotoxicity of 4-nonylphenol and nonylphenol ethoxylate mixtures by the use of Saccharomyces cerevisiae D7 mutation assay and use of this text to evaluate the efficiency of biodegradation treatments. Ecotoxicol Environ Saf 74: 253–258. 21087794 10.1016/j.ecoenv.2010.10.039

[34] Pupo M, Pisano A, Lappano R, Santolla MF, De Francesco EM, et al (2012) Bisphenol A Induces Gene Expression Changes and Proliferative Effects through GPER in Breast Cancer Cells and Cancer-Associated Fibroblasts. Environ Health Perspect 120: 1177–1182. 22552965 10.1289/ehp.1104526 PMC3440081

[35] Barton M (2012) The membrane estrogen receptor GPER - Clues and questions. Steroids 77: 935–942. 22521564 10.1016/j.steroids.2012.04.001

[36] Berner C, Aumüller E, Gnauck A, Nestelberger M, Just A, et al (2010) Epigenetic control of estrogen receptor expression and tumor suppressor genes is modulated by bioactive food compounds. Ann Nutr Metab 57: 183–189. 21088384 10.1159/000321514

[37] Anway MD, Rekow SS, Skinner MK (2008) Transgenerational epigenetic programming of the embryonic testis transcriptome. Genomics 91: 30–40. 18042343 10.1016/j.ygeno.2007.10.002 PMC2239263

[38] Liao HF, Tai KY, Chen WS, Cheng LC, Ho HN, et al (2012) Functions of DNA methyltransferase 3-like in germ cells and beyond. Biol Cell 104: 571–587. 22671959 10.1111/boc.201100109

[39] Sun MY, Yang XX, Xu WW, Yao GY, Pan HZ, et al (2012) Association of DNMT1 and DNMT3B polymorphisms with breast cancer risk in Han Chinese women from South China. Genet Mol Res 11: 4330–4341. 23079992 10.4238/2012.September.26.1

[40] Yang XX, He XQ, Li FX, Wu YS, Gao Y, et al (2012) Risk-association of DNA methyltransferases polymorphisms with gastric cancer in the southern chinese population. Int J Mol Sci 13: 8364–8378. 22942708 10.3390/ijms13078364 PMC3430239

[41] Borghese B, Santulli P, Héquet D, Pierre G, de Ziegler D, et al (2012) Genetic polymorphisms of DNMT3L involved in hypermethylation of chromosomal ends are associated with greater risk of developing ovarian endometriosis. Am J Pathol 180: 1781–1786. 22401780 10.1016/j.ajpath.2012.01.009

[42] Kristensen DG, Mlynarska O, Nielsen JE, Jacobsen GK, Rajpert-De Meyts E, et al (2012) Heterogeneity of chromatin modifications in testicular spermatocytic seminoma point toward an epigenetically unstable phenotype. Cancer Genet 205: 425–431. 22819380 10.1016/j.cancergen.2012.05.003

[43] Okamoto K (2012) Epigenetics: a way to understand the origin and biology of testicular germ cell tumors. Int J Urol 19: 504–511. 22375557 10.1111/j.1442-2042.2012.02986.x

[44] Ushida H, Kawakami T, Minami K, Chano T, Okabe H, et al (2012) Methylation profile of DNA repetitive elements in human testicular germ cell tumor. Mol Carcinog 51: 711–722. 21809391 10.1002/mc.20831

[45] Beyrouthy MJ, Garner KM, Hever MP, Freemantle SJ, Eastman A, et al (2009) High DNA methyltransferase 3B expression mediates 5-aza-deoxycytidine hypersensitivity in testicular germ cell tumors. Cancer Res 69: 9360–9366. 19951990 10.1158/0008-5472.CAN-09-1490 PMC2795063

[46] Biswal BK, Beyrouthy MJ, Hever-Jardine MP, Armstrong D, Tomlinson CR, et al (2012) Acute hypersensitivity of pluripotent testicular cancer-derived embryonal carcinoma to low-dose 5-aza deoxycytidine is associated with global DNA Damage-associated p53 activation, anti-pluripotency and DNA demethylation. PLoS One 7: e53003. 23300844 10.1371/journal.pone.0053003 PMC3531428

[47] Zhang X, Odom DT, Koo SH, Conkright MD, Canettieri G, et al (2005) Genome-wide analysis of cAMP-response element binding protein occupancy, phosphorylation, and target gene activation in human tissues. Proc Natl Acad Sci USA 102: 4459–4464. 15753290 10.1073/pnas.0501076102 PMC555478

[48] Hervouet E, Vallette FM, Cartron PF (2009) Dnmt3/transcription factor interactions as crucial players in targeted DNA methylation. Epigenetics 4: 487–499. 19786833 10.4161/epi.4.7.9883

[49] Garzon R, Liu S, Fabbri M, Liu Z, Heaphy CE, et al (2009) MicroRNA-29b induces global DNA hypomethylation and tumor suppressor gene reexpression in acute myeloid leukemia by targeting directly DNMT3A and 3B and indirectly DNMT1. Blood 113: 6411–6418. 19211935 10.1182/blood-2008-07-170589 PMC2710934

[50] Xue Q, Lin Z, Cheng YH, Huang CC, Marsh E, et al (2007) Promoter methylation regulates estrogen receptor 2 in human endometrium and endometriosis. Biol Reprod 77: 681–687. 17625110 10.1095/biolreprod.107.061804

[51] Wang Y, Yu Q, Cho AH, Rondeau G, Welsh J, et al (2005) Survey of differentially methylated promoters in prostate cancer cell lines. Neoplasia 7: 748–760. 16207477 10.1593/neo.05289 PMC1501885

[52] Imura M, Yamashita S, Cai LY, Furuta J, Wakabayashi M, et al (2006) Methylation and expression analysis of 15 genes and three normally-methylated genes in 13 Ovarian cancer cell lines. Cancer Lett 241: 213–220. 16303245 10.1016/j.canlet.2005.10.010

[53] Anway MD, Leathers C, Skinner MK (2006) Endocrine disruptor vinclozolin induced epigenetic transgenerational adult-onset disease. Endocrinology 147: 5515–5523. 16973726 10.1210/en.2006-0640 PMC5940332

[54] Minami K, Chano T, Kawakami T, Ushida H, Kushima R, et al (2010) DNMT3L is a novel marker and is essential for the growth of human embryonal carcinoma. Clin Cancer Res 16: 2751–2759. 20460473 10.1158/1078-0432.CCR-09-3338

[55] Bourc’his D, Bestor TH (2004) Meiotic catastrophe and retrotransposon reactivation in male germ cells lacking Dnmt3L. Nature 431: 96–99. 15318244 10.1038/nature02886

[56] Chédin F (2011) The DNMT3 family of mammalian de novo DNA methyltransferases. Prog Mol Biol Transl Sci 101: 255–285. 21507354 10.1016/B978-0-12-387685-0.00007-X

[57] Walton EL, Francastel C, Velasco G (2011) Maintenance of DNA methylation: Dnmt3b joins the dance. Epigenetics 6: 1373–1377. 22048250 10.4161/epi.6.11.17978

[58] Hashimoto H, Vertino PM, Cheng X (2010) Molecular coupling of DNA methylation and histone methylation. Epigenomics 2 657–669. 21339843 10.2217/epi.10.44 PMC3039846

