# Supplementary figures and images for: An Alkylphenol Mix Promotes Seminoma Derived Cell Proliferation through an ERalpha36-Mediated Mechanism
Source: PLoS One. 2013 Apr 23;8(4):e61758. doi: 10.1371/journal.pone.0061758 (PMC3634018; doi:10.1371/journal.pone.0061758)

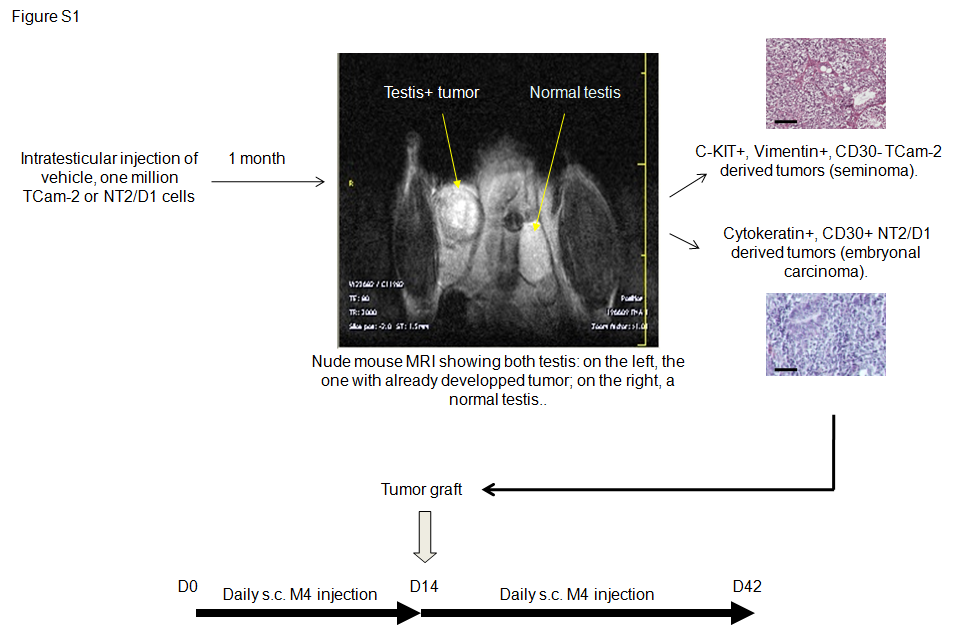

Supplement: Figure S1 — Characterization of testis tumor xenograft model in nude mice. Germ cell tumor xenograft models were first established after intra testicular injection of 1×10^7^ TCam-2 or NT2/D1 cells in 0,9% NaCl in nude mice. Tumors developed to approximately 0.5 cm3 in 6 weeks. MRI imaging was used (Spectro-imageur Bruker Biospec Avance 24/40; 2.4 teslas magnetic field) to confirm the presence of tumors into the scrotum. Tumors were harvested and seminoma or embryonal carcinoma identity was attested by histological and immunohistochemical analyses. The slices presented are hematoxylin/eosin/safran colorations of TCam-2 derived or NT2/D1 derived tumors. Tumor tissue was harvested and subcutaneously (s.c.) grafted in the inguinal pit of male nude mice. Because NT2/D1, but not TCam-2 derived tumors developed, we focused on the NT2/D1 model to examine the effects of M4 on tumor growth. Nude mice were s.c. implanted with 1–2 mm3 tumor pieces harvested from previously grown (0.5 cm3) NT2/D1 tumor (third passage). For alkylphenol assay, M4 or vehicle treatment was injected five days per week subcutaneously in male nude mice 2 weeks before, and 4 weeks after tumor graft in order to mimic everyday life contamination (see text for details). Bars are 100 µm long. (TIF) [file pone.0061758.s001.tif]

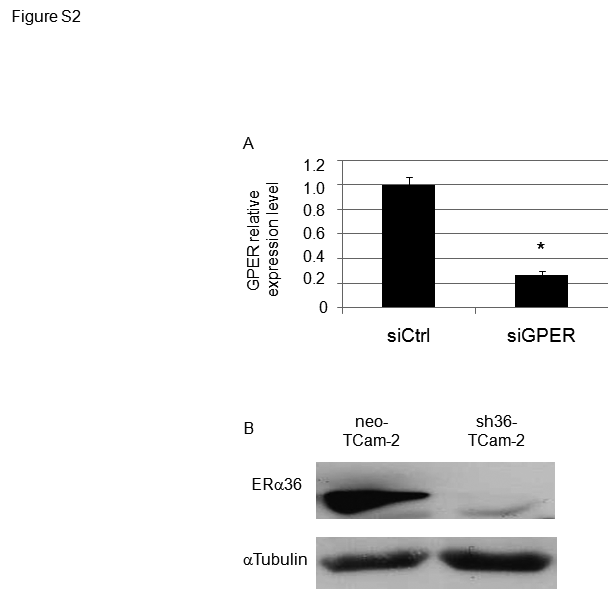

Supplement: Figure S2 — Real time PCR analysis (A) and western blot (B) showing the efficacy of GPER- or ERα36 -silencing in TCam-2 cells, respectively. (TIF) [file pone.0061758.s002.tif]

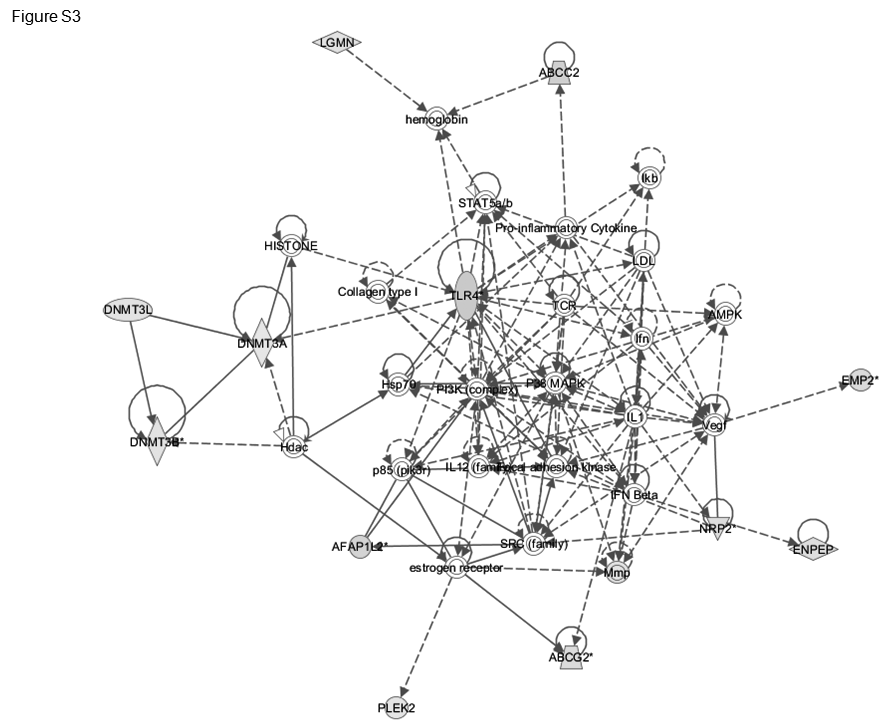

Supplement: Figure S3 — General scheme of Ingenuity software analysis showing the regulation network which involves DNMT3 gene family. (TIF) [file pone.0061758.s003.tif]
