# Supplementary material for: An Alkylphenol Mix Promotes Seminoma Derived Cell Proliferation through an ERalpha36-Mediated Mechanism
Source: PLoS One. 2013 Apr 23;8(4):e61758. doi: 10.1371/journal.pone.0061758 (PMC3634018; doi:10.1371/journal.pone.0061758)
Supplement: Table S1 — Primer and siRNA sequences. (DOCX) [file pone.0061758.s004.docx]

**Table S1:** Primer and siRNA sequences

|  | Gene | **Sequence** |
| --- | --- | --- |
| PCR primers | RPLPO | Fw 5'-GGCGACCTGGAAGTCCAACT-3’ |
|  |  | Rev 5'-CCATCAGCACCACAGCCTTC-3’ |
|  | DNMT3A | Fw 5'-CTGGGAGGAAGCGCAAGCAC-3’ |
|  |  | Rev 5'-CTGGGCCATGGATGGGGACT-3’ |
|  | DNMT3B | Fw 5'-CCAACAACACGCAACCAGTGG-3’ |
|  |  | Rev 5'-GCTGAGTCGTCAGCTGTGCG-3’ |
|  | DNMT3L | Fw 5'-CCATCCCAGATGTCCACGGC-3’ |
|  |  | Rev 5'-CAGAGCCCAGTGCCTGCTG-3’ |
| siRNA duplexes | GPER | Dup.1 5’-GCACCUUCAUGUCGCUCUU-3’ |
|  |  | Dup.2 5’-UUGAACACCUCAAUGAGGG-3’ |
|  |  | Dup.3 5’-AAGACUGCUUGCAGGGAGC-3’ |
