# Supplementary material for: An Alkylphenol Mix Promotes Seminoma Derived Cell Proliferation through an ERalpha36-Mediated Mechanism
Source: PLoS One. 2013 Apr 23;8(4):e61758. doi: 10.1371/journal.pone.0061758 (PMC3634018; doi:10.1371/journal.pone.0061758)
Supplement: Table S2 — Main results from Ingenuity analysis: top five networks in which M4 regulated genes are involved. (DOCX) [file pone.0061758.s005.docx]

**Table S2 :** Main results from Ingenuity analysis : top five networks in which M4 regulated genes are involved.

| **Top networks** | **Score**  [28] |
| --- | --- |
| Cancer , Cellular movement, Tumor morphology | 35 |
| Developmental disorder | 31 |
| Cellular growth and proliferation | 38 |
| Cancer, Tumor morphology, Connective tissue disorders | 27 |
| Cell death and survival, Hereditary disorder | 27 |
