# Supplementary material for: An Alkylphenol Mix Promotes Seminoma Derived Cell Proliferation through an ERalpha36-Mediated Mechanism
Source: PLoS One. 2013 Apr 23;8(4):e61758. doi: 10.1371/journal.pone.0061758 (PMC3634018; doi:10.1371/journal.pone.0061758)
Supplement: Table S3 — Main results from Ingenuity analysis: biological functions in which M4 regulated genes are involved. (DOCX) [file pone.0061758.s006.docx]

**Table S3 :** Main results from Ingenuity analysis : biological functions in which M4 regulated genes are involved.

| **Biological functions**  (classified by p-value: 6.56E-09 < p-value < 8.95E-03) | | **Number of genes** |
| --- | --- | --- |
| **Diseases and disorders** | Cancer (invasion and development) | 79 |
|  | Endocrine system disorders | 21 |
|  | Reproductive system disease | 40 |
| **Molecular and cellular functions** | Cellular growth and proliferation | 66 |
|  | DNA replication, Recombination and Repair | 4 |
|  | Gene expression | 4 |
|  | Cellular movement | 28 |
|  | Cellular development | 30 |
| **Physiological System Development and Function** | Tumor morphology | 26 |
|  | Endocrine system development and function | 19 |
|  | Organ development | 51 |
|  | Renal and urological system development and function | 18 |
