# Supplementary material for: An Alkylphenol Mix Promotes Seminoma Derived Cell Proliferation through an ERalpha36-Mediated Mechanism
Source: PLoS One. 2013 Apr 23;8(4):e61758. doi: 10.1371/journal.pone.0061758 (PMC3634018; doi:10.1371/journal.pone.0061758)
Supplement: Table S4 — Main results from Ingenuity analysis: Top five predicted upstream regulators of M4 regulated genes. (DOCX) [file pone.0061758.s007.docx]

**Table S4 :** Main results from Ingenuity analysis: Top five predicted upstream regulators of M4 regulated genes.

| **Predicted upstream regulator** | **P-value of overlap** |
| --- | --- |
| beta-estradiol | 6.01E-08 |
| ERBB2 | 1.13E-07 |
| ESR1 | 4.11E-07 |
| dexamethasone | 1.20E-06 |
| diethylstilbestrol | 1.43E-06 |
