# Supplementary material for: An Alkylphenol Mix Promotes Seminoma Derived Cell Proliferation through an ERalpha36-Mediated Mechanism
Source: PLoS One. 2013 Apr 23;8(4):e61758. doi: 10.1371/journal.pone.0061758 (PMC3634018; doi:10.1371/journal.pone.0061758)
Supplement: Table S5 — Top list of the 20 genes deregulated by M4 after 1 hour or 24 hour exposure. (DOCX) [file pone.0061758.s008.docx]

**Table S5:** Top list of the 20 genes deregulated by M4 after 1 hour or 24 hour exposure.

|  |  | 1h | |  |  |  | 24h | |
| --- | --- | --- | --- | --- | --- | --- | --- | --- |
| Transcript | Gene | p -value | Variation |  | Transcript | Gene | p -value | Variation |
| NM_013940 | OR10H1 | 0,02 | 10,29 |  | NM_020844 | KIAA1456 | 0,00 | 6,87 |
| NM_000569 | FCGR3A | 0,01 | 6,85 |  | NM_013940 | OR10H1 | 0,00 | 5,99 |
| XM_931923 | EBF2 | 0,00 | 5,70 |  | BC036192 | COL14A1 | 0,02 | 5,56 |
| BC017865 | FCGR3A | 0,02 | 4,90 |  | NM_016591 | GCNT4 | 0,03 | 4,69 |
| XM_933727 | LOC440345 | 0,01 | 4,87 |  | BC017865 | FCGR3A | 0,02 | 4,06 |
| XM_931500 | LOC643359 | 0,03 | 4,54 |  | NM_173663 | NY-REN-7 | 0,01 | 3,78 |
| AK125579 | N/A | 0,01 | 4,51 |  | BC015413 | ROPN1B | 0,00 | 3,77 |
| BC093880 | SERF1A | 0,01 | 4,50 |  | NM_001004693 | OR2T10 | 0,02 | 3,62 |
| NM_001004693 | OR2T10 | 0,01 | 4,41 |  | NM_004770 | KCNB2 | 0,01 | 3,60 |
| BC067423 | B3GNT3 | 0,01 | 4,17 |  | NM_138969 | RDHE2 | 0,02 | 3,52 |
| NM_001012966 | KLK6 | 0,04 | 0,21 |  | BC063118 | DIO2 | 0,01 | 0,21 |
| NM_015053 | PPFIA4 | 0,01 | 0,21 |  | NM_001012966 | KLK6 | 0,00 | 0,21 |
| BC063118 | DIO2 | 0,01 | 0,21 |  | NM_014379 | KCNV1 | 0,01 | 0,21 |
| NM_001001936 | KIAA1914 | 0,04 | 0,20 |  | BC069353 | HAS2 | 0,00 | 0,20 |
| BC028739 | KCNV1 | 0,04 | 0,18 |  | BC109071 | HAS2 | 0,00 | 0,19 |
| NM_014379 | KCNV1 | 0,01 | 0,18 |  | NM_005328 | HAS2 | 0,01 | 0,18 |
| BC069353 | HAS2 | 0,00 | 0,17 |  | NM_153225 | RPESP | 0,00 | 0,18 |
| BC099646 | NAALAD2 | 0,03 | 0,17 |  | X75299 | VIPR1 | 0,01 | 0,16 |
| NM_005328 | HAS2 | 0,01 | 0,17 |  | BC045606 | NID1 | 0,01 | 0,15 |
| BC109071 | HAS2 | 0,00 | 0,16 |  | NM_004624 | VIPR1 | 0,00 | 0,15 |
| BC046645 | CCBE1 | 0,03 | 0,15 |  | NM_005211 | CSF1R | 0,03 | 0,15 |
| BC045606 | NID1 | 0,00 | 0,14 |  | NM_003982 | SLC7A7 | 0,03 | 0,14 |
| BC038840 | NAALAD2 | 0,05 | 0,14 |  | BC064424 | VIPR1 | 0,00 | 0,11 |
